# Tumor type and cell type-specific gene expression alterations in diverse pediatric central nervous system tumors identified using single nuclei RNA-seq

**DOI:** 10.21203/rs.3.rs-2517703/v1

**Published:** 2023-02-23

**Authors:** Min Kyung Lee, Nasim Azizgolshani, Joshua A. Shapiro, Lananh N. Nguyen, Fred W. Kolling, George J. Zanazzi, Hildreth Robert Frost, Brock C. Christensen

**Affiliations:** 1 Department of Epidemiology, Geisel School of Medicine at Dartmouth, Lebanon, NH, USA; 2 Department of Cardiothoracic Surgery, Columbia University Irving Medical Center, New York, NY, USA; 3 Childhood Cancer Data Lab, Alex’s Lemonade Stand Foundation, Bala Cynwyd, PA, USA; 4 Department of Laboratory Medicine and Pathobiology, University of Toronto, Toronto, ON, Canada; 5 Dartmouth Cancer Center, Lebanon, NH, USA; 6 Department of Pathology and Laboratory Medicine, Geisel School of Medicine at Dartmouth, Lebanon, NH, USA; 7 Department of Biomedical Data Science, Geisel School of Medicine at Dartmouth, Lebanon, NH, USA; 8 Department of Molecular and Systems Biology, Geisel School of Medicine at Dartmouth, Lebanon, NH, USA; 9 Department of Community and Family Medicine, Geisel School of Medicine at Dartmouth, Lebanon, NH, USA

## Abstract

Central nervous system (CNS) tumors are the leading cause of pediatric cancer death, and these patients have an increased risk for developing secondary neoplasms. Due to the low prevalence of pediatric CNS tumors, major advances in targeted therapies have been lagging compared to other adult tumors. We collected single nuclei RNA-seq data from 35 pediatric CNS tumors and three non-tumoral pediatric brain tissues (84,700 nuclei) and characterized tumor heterogeneity and transcriptomic alterations. We distinguished cell subpopulations associated with specific tumor types including radial glial cells in ependymomas and oligodendrocyte precursor cells in astrocytomas. In tumors, we observed pathways important in neural stem cell-like populations, a cell type previously associated with therapy resistance. Lastly, we identified transcriptomic alterations among pediatric CNS tumor types compared to non-tumor tissues, while accounting for cell type effects on gene expression. Our results suggest potential tumor type and cell type-specific targets for pediatric CNS tumor treatment. In this study, we address current gaps in understanding single nuclei gene expression profiles of previously uninvestigated tumor types and enhance current knowledge of gene expression profiles of single cells of various pediatric CNS tumors.

## Introduction

Central nervous system (CNS) tumors account for ~25% of pediatric cancer cases and are the leading cause of cancer death in children and adolescents in the United States^1^. Incident pediatric CNS tumors are comprised of many histologically distinct tumor types including pilocytic astrocytomas (15.2%), embryonal tumors (9.4%), and neuronal/mixed neuronal-glial tumors (7.9%)^2^. Survival rates vary widely among tumor types, with a good 10-year survival of 95.4% for pilocytic astrocytomas and a poor 10-year survival of 15.9% for pediatric high-grade gliomas^2^. Pediatric CNS tumor patients are at risk of developing secondary neoplasms, with a 30-year cumulative incidence of malignant secondary neoplasms ranging from 4.7 – 7.8%^3,4^. The standard of care treatments for primary CNS tumors include surgery, radiotherapy, and chemotherapy with relatively limited options for targeted therapy compared to tumors in other anatomic regions.

Recent advances in identifying molecular subtypes in various pediatric CNS tumor types have been made utilizing genomic, transcriptomic and epigenomic data as reflected in the 2021 World Health Organization classification of CNS tumors^5^. For example, medulloblastoma can be classified into four separate molecularly defined subtypes: WNT-activated, SHH-activated and *TP53*-wildtype, SHH-activated and *TP53*-mutant, and non-WNT/non-SHH^6-10^. In addition, supratentorial ependymoma can be categorized into *ZFTA* fusion-positive or *YAP1* fusion-positive^11,12^. A better understanding of the molecular variations that exist even among each tumor type has led to novel treatment options. For example, Larotrectinib and entrectinib, targeted therapies for *NTRK*fusion, which has been found in brain tumors, have been approved by the Food and Drug Administration to treat some brain tumors that are metastatic or unresectable with surgery^13,14^.

In addition to the molecular characterization of bulk pediatric CNS tumor tissue, emerging work has begun to investigate the transcriptome and cellular states that exist in these tumors at the single cell level. One of the first single cell transcriptomics contributions focused on *H3K27M*-altered pediatric gliomas (n=6, and 3,300 cells) showed that tumors are mainly composed of progenitor cell-like oligodendrocyte populations, rather than differentiated malignant cells^15^. Later, Gojo et al. identified that cellular hierarchies in primary ependymomas (n=28) reflect impaired neurodevelopment and that undifferentiated programs can infer prognosis^16^. Moreover, Gillen et al. revealed that subpopulations in ependymomas (n=26) impact tumor molecular classification of bulk transcriptomes^17^. In medulloblastomas (n=25 and 9,000 cells), Hovestadt et al. identified specific subpopulations associated with molecular subtypes^7^. For example, Group 4 medulloblastoma are composed of differentiated neuronal-like neoplastic cells, while the other three groups are composed of subgroup-specific undifferentiated and differentiated neuronal-like malignant populations^7^.

While these single cell and single nucleus transcriptomics studies in 85 total primary CNS tumors to date have improved our understanding of cell states in pediatric CNS tumors, there is still much to be investigated to advance optimal therapeutic options for both primary cancer treatment and reduction of secondary neoplasms. Due to limited sample availability for these rare pediatric CNS tumors, progress in single cell level characterization of these tumors has been relatively slow. Here, we characterized single nuclei gene expression profiles of 35 pediatric CNS tumors and 3 non-tumor pediatric brain tissues. Our study augments previous studies by incorporating single nuclei gene expression profiles of additional pediatric CNS tumor types (dysembryoplastic neuroepithelial tumors, gangliogliomas, etc.) and non-tumor pediatric brain tissue which have not yet been published to our knowledge.

## Results

Samples from pediatric central nervous system tumors and non-tumor pediatric brain tissue were obtained from patients being treated at Dartmouth-Hitchcock Medical Center and Dartmouth Cancer Center from 1993 to 2017. Non-tumor pediatric brain tissues from the supratentorial regions were collected from patients undergoing surgical resection for epilepsy. Patient characteristics are described in **Table 1**. Pathological re-review for histopathologic tumor type and grade were done according to the 2021 World Health Organization CNS tumor classification system and categorized into the broader tumor types to balance sample size per tumor type^5^. Specific diagnoses for each sample can be found in **Supplementary Table 1.**

Genetic variants were identified using bulk tissue RNA-seq data for all tumors except for two tumors due to low bulk RNA-seq data quality. Copy number variations (CNV) were determined using bisulfite treated DNA methylation array data. Genetic and cytogenic variations varied among tumors and tumor types (**Figure 1**). Interestingly, across all but one tumor sample, tumors had genetic variants in *MALAT1*. Many of the genetic variants detected within the pediatric CNS tumors were associated with epigenetic processes. For example, almost half of the tumors, across tumor types, had genetic variants in *HIST1H1E* (14/33). CNV patterns in some tumor types were as expected from previous literature. For instance, 5 out of the 9 ependymoma had chromosome 1q gain, which has been considered to be an early tumorigenic event in ependymoma^18,19^.

### Integrated de-multiplexing method to increase single nuclei RNA-seq data yield

Using lipid-tagged hashtag oligonucleotides (HTO), 34 samples (out of 38 total samples) were multiplexed in 17 pools to collect 10X genomics snRNA-seq data^20^. The distribution of samples across sequencing runs and pools is provided in **Supplementary Table 1.** As many nuclei were not tagged with sufficient HTO to be efficiently demultiplexed in downstream analyses, we aimed to augment demultiplexing by analyzing sequencing-derived genotype data from each nucleus together with HTO information and assign additional nuclei to specific samples (**Figure 2A**). To summarize our demultiplexing process, we first used HTOs to assign the nuclei to their respective samples. For samples that were assigned confidently with the HTO, we filtered to keep only the nuclei that were assigned to the same sample concordantly using genotype information. For nuclei that were either unassigned to a sample or assigned as a doublet with HTO, we assigned nuclei to samples using genotype information (detailed in the methods section). The final set of nuclei per sample were comprised of the filtered nuclei from HTO and genotype identified nuclei. An example of the single nucleotide variants identified per pool along with their assigned sample can be found in **Figure 2B**. An example of how many nuclei were obtained for one pool, during each step, is shown in **Figure 2A** on the right. The integrated demultiplex method classified an average of 1,921 nuclei per sample (range = 234 – 5795, **Table 1**). The number used in downstream analysis per sample is included in **Supplementary Table 1**. The total number of demultiplexed nuclei was increased 47.4% (additional 27,248 nuclei) using the integrated approach over the HTO-only method, and 15.6% (additional 11,445 nuclei) over the genotype-based method alone (**Figure 2C**). Gene expression profiles for a total of 84,700 nuclei were used for downstream analyses.

### Cell type heterogeneity in pediatric central nervous system tumors and non-tumor pediatric brains

Out of 84,700 nuclei, 67,249 nuclei (79%) were from pediatric CNS tumors and 17,451 nuclei (21%) were from non-tumor tissue (**Figure 3A**). Across all samples, snRNA-seq data revealed 58 clusters that were grouped into 16 major cell types: astrocytes (AST), embryonal tumor cells (EMB), endothelial cells (EN), macrophage/microglia (MAC/MG), neurons (NEU), excitatory neurons (NEU_EX), granular neurons (NEU_GN), inhibitory neurons (NEU_INH), interneurons (NEU_INT), neural stem cells (NSC), oligodendrocytes (OLIG), oligodendrocyte precursor cells (OPC), radial glial cells (RGC), stromal cells (ST), T cells (TC), and unipolar brush cells (UBC) (**Supplementary Figure 1A, Figure 3B**). The clusters were classified into cell types using classical markers for cell types found in the brain (**Table 2**). The following gene markers for cell types were used: *GFAP* and *AQP4* for astrocytes; *FN1* and *COL4A1* for endothelial cells; *CSF1R* and *PTPFIC* for macrophage/microglia; *RBFOX3* and *RELN* for neurons and unipolar brush cells; *GAD2* for inhibitory neurons and interneurons; *SOX2* and *CD44* for neural stem-like cells; *MOG* and *PLP1* for oligodendrocytes; *PDGFRA* for oligodendrocyte precursor cells; *VIM, NES*, and *PAX6* for radial glial cells; *FAP* for stromal cells; *CD3E* for T cells. Not all gene markers corresponded to expected expression levels for the major cell types. For example, the neural stem cells (NSCs) did not express classical neural stem cell like genes (*SOX2* and *CD44*) but were identified by enrichment testing of neural stem cell/neural progenitor-like cell gene sets. Because the embryonal tumor cells (EMB) clusters were unlike any other classical cell type found in the brain, the cells in these clusters were classified as embryonal tumor cells. These marker-based cell type classifications were subsequently validated by enrichment of cell type-specific pathways using the Variance-adjusted Mahalanobis method, a single cell-level pathway enrichment method (**Figure 3C, Supplementary Figure 1B)**^21-28^. The cell type-specific pathways used for enrichment testing were derived from single cell RNA-seq experiments of developing human and mouse brains.

To identify stem-like phenotypes in our tumor nuclei population, we investigated the expression levels of classically used markers of cancer stem cells (*ITGA6, CD44, PROM1, NES, MSI1, MYC, NANOG, SOX1, SOX2, POU5F1, VIM, SDC1, SDC2, GPC1, GPC2*), as well as an enrichment score for stemness from Tirosh et al^29,30^. Levels of expression for genes classically used for to isolate stem-like cells in literature varied among the different cell types (**Supplementary Figure 2**). Interestingly, cell types expected to be more differentiated, like astrocytes, had relatively high levels of *CD44* and *VIM,* and these genes were expressed in many of the cell types. In addition, although the NSC-like cluster had a high stemness score, the expression of cancer stem cell markers was minimal. Unexpectedly, the UBC-like clusters also had elevated stemness scores. While gene expression levels may not always correlate with protein expression, our results indicate cell types identified using classical stem cell markers may not capture all tumor cells with stemness features.

Next, we tested for potential associations of clinical variables with tumor stemness scores. We first assessed the distribution of stemness scores among nuclei in each sample and determined the median stemness score (**Supplementary Figure 3**). We found that the stemness scores were higher in embryonal compared to other tumor types or non-tumor tissue (**Supplementary Figure 4A**). Specifically, embryonal tumors had significantly higher stemness scores compared to astrocytomas (*P*-value = 0.03), ependymoma (*P*-value = 0.02), and glioneuronal/neuronal tumors (*P*-value = 0.029). Compared with low grade tumors, high grade tumors had higher stemness scores (*P*-value = 0.008, **Supplementary Figure 4B**), and somewhat unexpectedly, stemness score was positively correlated with age (R = 0.47, *P*-value = 0.004, **Supplementary Figure 4C**). No difference in stemness score was observed between tumors in the subtentorial and supratentorial regions of the brain (*P*-value = 0.600, **Supplementary Figure 4D**). Our results indicate that stemness level of single cells is associated with tumor type and grade, which may be important when considering potential for therapy resistance and metastasis and when developing targeted therapies.

To reveal any specific cell populations that are only present in a restricted set of tumor types, we evaluated the association between cell type proportions and tumor type (**Figure 4, Supplementary Figure 5**). Non-tumor tissue contained nuclei from all major expected cell types found in normal brain, including astrocytes, oligodendrocytes, and excitatory and inhibitory neurons which demonstrated the high-quality data derived from the non-tumor tissues. Some tumor samples had small proportions of cell types normally present only in non-tumor tissue, such as excitatory neuron cluster #5 (NEU_EX5) and inhibitory neuron cluster #2 (NEU_INH2). These cases are likely the result from the inclusion of cells from the tumor margin. Non-tumor tissues had limited numbers of nuclei from progenitor-like cell types, like NSCs, RGCs, or UBCs. While OPCs are a progenitor cell type, they are also found in normal brain tissue. The non-tumor OPCs were limited to the OPC4, a population transcriptionally distinct from tumor OPCs residing in OPC1-3.

Some cell types were exclusive to a specific tumor. For example, the glioblastoma sample was comprised of 91% NSC1, and an ependymoma sample consisted predominantly (86%) of OPC2. MG2 was present at higher proportions (mean = 3.3%, range = 0.3 – 31.2%) in tumors compared to non-tumor tissue (0.9%). All astrocytomas had at least small proportions of A4, OPC1, and OPC5. The embryonal tumors had cell types that were more neuronal (apart from EMB cell types) like NSCs and UBCs. Large proportions of ependymoma samples were made of RGC clusters. The glioneuronal/neuronal tumor type samples were more varied in terms of which cell types were more present in each tumor. The expanded cell types were consistent with some known cell types of origin for these tumors, such as the RGCs in the ependymomas.

### Cell type-specific pathway enrichment in pediatric CNS tumors

First, to determine cell type-specific pathway enrichment in each nuclei of the tumor samples, we conducted a pathways analysis at the single cell level using the Variance-adjusted Mahalanobis (VAM) method, which computes cell-level pathway scores that account for the technical noise and inflated zero counts of single cell RNA-seq data^21^. We used 196 pathways from the MSigDB Pathway Interaction Database (PID) collection for our enrichment testing^23,24,31^. The cell-level enrichment p-values generated by VAM were corrected for false discovery rate using the Benjamini-Hochberg method and classified to be significantly enriched in each nucleus if the FDR adjusted p-value was less than 0.1 as binary classifications (enriched or not enriched).

Next, we determined any pathways that were more specific for each cell type to determine pathways important in each cell type. The PID pathways were considered to be important/specific to the cell type under adjusted p-value < 0.05 threshold in the differential enrichment test. For cell types with a limited presence in tumor tissues, like many of the excitatory neurons and A1, we observed no pathways that were specific to the clusters (**Supplementary Figure 6A, Supplementary Table 2**). The immune-related cells (MG1, MG2, and TC), which were present in tumor tissue at slightly higher levels than in non-tumor tissue, had more than 44% of the PID pathways specific to these cell types. The high percentage of PID pathways that were important in the immune-related cell types is likely due to the relatively greater number of cytokine and other immune-associated pathways are included in the PID database.

All NSC clusters, except for NSC6 (3.6% pathways), had more than 10% of PID pathways that were important to the NSCs (range = 11.73 – 42.35, **Supplementary Figure 6A, Supplementary Table 2**). While there were no shared pathways that were considered to be important in all 8 NSC clusters, there were numerous pathways shared among majority of the NSCs (**Figure 5A, Supplementary Figure 6B**). The retinoic acid pathway and telomerase pathway were considered to be important in 7 of 8 NSC clusters (**Figure 5B**). Aurora-B, PLK1, FOXM1, E2, ATR, FOXO, Retinoic Acid pathways were considered to be important in just 6 of the 8 NSC clusters. Our results provided potential cell type-specific targets within these PID pathways important for each cluster for future therapeutic strategies.

### Transcriptomic alterations in tumors compared to non-tumor at the single cell level

We next aimed to determine transcriptomic alterations in pediatric CNS tumors compared to non-tumor pediatric brain tissue. In bulk differential gene expression analyses, it is typically not possible to account for the impact of cell composition differences on gene expression levels^32-35^. Here, using single nuclei level data, we compared expression of the 4,000 most variable genes in nuclei from each tumor type to the gene expression of nuclei in non-tumor tissue, controlling for cell-type composition differences (**Figure 6A**). Genes were considered differentially expressed if they met the FDR < 0.05 threshold.

As expected, adjusting for cell type proportions reduced the number of significantly differentially expressed genes compared with cell-type-unadjusted analyses. However, importantly, cell-type-adjusted analyses identified on average 200 genes per tumor type that not observed in unadjusted models. (**Figure 6B, Supplementary Figure 7A, 7B, Supplementary Table 3**). Moreover, cell type-unadjusted analyses identified average 758 genes per tumor types that were not detected in adjusted models. Genes uniquely identified in cell-type-adjusted models represent underlying tumor biology that was obscured by variation in cell type proportions composing the tumor microenvironment across subjects (**Figure 6C**). For example, *WNT3A,* a gene shown to mediate glioblastoma progression^36^ was shown to be upregulated in glioneuronal/neuronal tumors and Schwannoma only using the adjusted analysis (**Supplementary Table 3**). Furthermore, the unadjusted model often gave estimates that were contrary to the direction of change from the adjusted model. For example, *FAT2* was significantly decreased (estimate = −1.80) in embryonal tumors relative to non-tumor tissue in the adjusted model but significantly increased (estimate = 0.42) in embryonal tumors in the unadjusted model. Also, *FGFR2* had significantly increased in expression (estimate = 0.76) in the Schwannoma nuclei relative to non-tumor tissue in the adjusted model but was significantly decreased in expression (estimate = −0.52) in the unadjusted model.

Using cell type-adjusted models, we detected tumor type-specific alterations in gene expression compared to non-tumor tissue. In astrocytomas, we identified 958 significantly downregulated and 970 significantly upregulated genes compared to non-tumor tissue (FDR < 0.05). Genes upregulated in astrocytomas include *ID4, CD74* and *FOS*. The differentially expressed (DE) genes in astrocytomas were associated with translation-related and nonsense-mediated decay-related processes (**Supplementary Figure 8A, Supplementary Table 4**). Embryonal tumors had 915 downregulated and 944 upregulated genes relative to non-tumor tissue that were associated with rRNA processing and translation-associated processes (**Supplementary Figure 8B, Supplementary Table 5**). In embryonal tumors, the topmost DE genes included many ribosome-associated genes like *RPS2, RPLP1,* and *RPL13A* as well as histone H3.3 related genes like *H3F3A* and *H3F3B*. Ependymomas had 1024 downregulated and 1213 upregulated genes compared to non-tumor tissue. The topmost DE genes were *IGFBP5, CFAP54* and *COLEC12*. Similar to astrocytomas, DE genes in ependymomas were associated with translation and nonsense-mediated decay related processes (**Supplementary Figure 8C, Supplementary Table 6**). Glioneuronal/neuronal tumors had 1,035 downregulated and 1,079 upregulated genes relative to non-tumor tissue; these genes that were associated with extracellular matrix and integrin-related processes and MET signaling (**Supplementary Figure 8D, Supplementary Table 7**). *TAFA1, ALK,* and *VAV3* were some of the topmost DE genes in glioneuronal/neuronal tumors. In the glioblastoma, there were 1,575 downregulated genes and 524 upregulated genes that were associated with RNA processing and translation-related processes (**Supplementary Figure 8E, Supplementary Table 8**). Some genes that were topmost DE in glioblastoma nuclei include *RMST, ID4* and *PBX3*. Lastly, in the Schwannoma, there were 864 downregulated genes and 813 upregulated genes relative to non-tumor tissue that were associated with elastic fibers and RHO/RAC1 GTPases cycles (**Supplementary Figure 8F, Supplementary Table 9**). *CEMIP, THSD4,* and *GPC6* were among the topmost DE genes in the Schwannoma nuclei. The list of differentially expressed genes and their associated pathways per tumor type are listed in **Supplementary Table 3 – 9**.

Of the 4,000 most variable genes that were used in differential gene expression analysis, there were 558 genes that were differentially expressed in all six of the tumor types, 717 in five of the tumor types, and 596 in four of the tumor types compared with non-tumor tissue (**Figure 7A, Table 3**). There were differentially expressed genes specific to a single tumor type: 43 genes for astrocytomas, 61 for embryonal tumors, 52 for ependymomas, 68 for glioneuronal/neuronal tumors, 98 for glioblastoma, and 57 for Schwannoma. While 60.9% (340/558) of the differentially expressed genes shared among all the tumor types were either increased or decreased the same direction, the remainder of genes varied in the direction of change based on tumor type compared to non-tumor tissue. The proportion of genes that either increased or decreased in the same direction for the shared significantly differentially expressed among all tumor types were significantly higher than expected (*P-value* < 2.2x10^−16^). Protein-coding genes with increased expression across all tumor types included *E2F7, ETS1, EZH2, ID3/4, MKI67, PIK3R3,* and *TOP2A*. We conducted a pathways analysis of the genes with increased expression across all tumor types, and genes with decreased expression across all tumor types with Reactome pathways^37^. Interestingly, translation or nonsense mediated decay related processes having increased expression across all tumor types compared to non-tumor tissues (**Figure 7B**). Shared decreased protein-coding genes across all tumor types included *FOXP2, GABRA1/2/4/5, NRGN, SST,* and *SYNPR*. Even when differential gene expression analyses were adjusted for cell type, across all tumor types, there was decreased expression in genes associated with neuronal system such as transmission across chemical synapses and activation of NMDA or GABA receptors (**Figure 7C**). Hierarchical clustering of the differentially expressed genes revealed that transcriptomic alterations were similar in ependymomas and glioneuronal/neuronal tumors and likewise in astrocytomas and embryonal tumors (**Figure 7A**).

## Discussion

In this study, we characterized gene expression profiles of 84,700 nuclei from snRNA-seq of 35 pediatric CNS tumors and 3 pediatric non-tumor brain tissues. We utilized an integrated hashtag oligonucleotide and genotype-based methods to maximize the number of sample-assigned nuclei from our multiplexed snRNA-seq experiment. Although the original MULTI-seq^20^ work showed that multiplexing nuclei was feasible, some difficultly encountered with the approach in our study may have been attributable to use of fresh frozen samples that had been stored in the freezer for decades. In our study, we detail a novel approach to increase the number of cells assigned to a specific sample from pooled sequencing runs by integrating a genotype-based approach to demultiplex snRNA-seq data. Future studies are expected to benefit from our integrated demultiplexing method to maximize data usage while decreasing the cost of snRNA-seq experiments.

Our study incorporates pediatric CNS tumor types that have not yet been characterized with single cell or single nuclei RNA-seq such as gangliogliomas. Moreover, we incorporated non-tumor pediatric tissues in our experiment, which to our knowledge have not been included in previous pediatric CNS tumor single cell RNA-seq studies. We describe changes in cell type proportions specific to each tumor type and use this information to identify the gene expression profiles and pathways enriched across tumor and normal samples through a cell type-adjusted analysis.

We characterized major cell subpopulations in specific tumor types, some of which have not been previously established. This includes the expansion of oligodendrocyte precursor cell (OPC) subpopulations in astrocytomas, and unipolar brush-like cells (UBC) with high stemness levels enriched in embryonal tumors. In the ependymomas, there was a significant presence of radial glial-like cells (RGC). Some glioneuronal/neuronal tumors featured stromal cells (ST) that were less present in other tumor types, demonstrating significant variability even within subtypes of tumors. The glioblastoma sample was predominantly comprised of a neural stem cell-like cell population. The Schwannoma sample was comprised of a specific stromal cell type. Despite some overlap in the major cell types between tumor and non-tumor nuclei, their gene expression profiles were distinct. For example, the OPC4 cluster is unique to non-tumor nuclei, while tumor OPCs reside in OPC1-3. Some neuron-like clusters (i.e. NEU_EX3) that were present in tumors had very limited presence in the non-tumor samples. Our results suggest distinct tumor-associated gene expression alterations even if the tumor cell may resemble a normal brain cell type.

Our study supported some key findings from previous scRNA-seq experiments in ependymomas. Gojo et al along with other studies identified radial glial like cells as potential cells of origin in ependymomas^16,38,39^. Our results corroborate this finding with an abundance of radial glial cells in our ependymoma samples. Moreover, Gojo et al indicate that stem-like cell populations are associated with more aggressive ependymomas^16^. Our results indicate a similar pattern in our expanded pediatric CNS tumor types, in which higher grade tumors are associated with cells with more stemlike features. Our study also supported results from Reitman et al, who demonstrated that pilocytic astrocytoma tumors are overall comprised of OPCs and mature glial-like cells^40^. Our results indicated a similar pattern in which much of our pilocytic astrocytoma samples were comprised of varying OPC clusters and couple of astrocyte-1 ike clusters. The similarity of our results with previously published studies supports our results and previous findings in separate patient populations.

We identified the pathways enriched in varying cell types, with a focus on neural stem like cells. Since NSCs have been shown to be associated with therapy resistance, metastasis, and tumor malignancy, it is important to specifically consider NSCs when treating pediatric CNS tumors and reducing risk for secondary neoplasms^41-47^. We determined potential targetable NSC-specific pathways. While some commonly enriched pathways like MYC and FOXM1 in NSCs may be considered very difficult to target as MYC and transcription factors are considered to be less druggable, there were more easily targetable pathways enriched in NSCs like Aurora-B kinase and retinoic acid pathway.

With our cell type-adjusted approach, we addressed a critical confounder in differential gene expression analyses to identify transcriptomic alterations that exist in tumors compared to non-tumor tissue. Although the number of significantly differentially expressed genes decreased in the cell type-adjusted model compared to the cell type-unadjusted model, the adjusted model identified novel genes associated with tumors that would not have been uncovered in the unadjusted model. Moreover, the significantly differentially expressed genes exclusive to the unadjusted model likely stem from variations in cell type proportions, rather than from the underlying tumor biology that would be necessary for discovering effective therapeutic targets.

The pathways associated with the differentially expressed genes across the multiple tumor types in the cell type-adjusted model (translation associated processes like peptide chain elongation and translation initiation/termination along with nonsense mediated decay (NMD) processes) suggest the importance of these pathways commonly being dysregulated in pediatric central nervous system tumors. Previous studies have suggested the importance of downregulation of NMD responses in the differentiation of neural stem cells^48-50^. Moreover, high levels of NMD factors were sufficient to keep the stemness of neural stem cells^48^. Interestingly, our results indicate upregulation of NMD associated genes across all pediatric CNS tumor types in comparison to non-tumor pediatric brain which suggest the potential mechanism of upregulation of NMD maintaining more stem-like cells in these tumors. As more stem-like cells contributes to therapy resistance and recurrence, further studies investigating the NMD pathways and how they can be exploited to be potential therapeutic targets in pediatric CNS tumors are necessary.

Our study characterizes the heterogeneity that exists across pediatric CNS tumor types in comparison to non-tumoral pediatric brain tissue at the single cell level. We also identify potential tumor type and cell type-specific molecular characteristics that may be used therapeutic targets for the various pediatric CNS tumors from primary tissue samples. Although there were very limited samples for Schwannomas and glioblastoma, our study included thousands of nuclei from these tumor types to gain a better understanding of cells that exist in these tumor types that previous studies have not investigated yet. From our results, complementary preclinical *in vitro* and *in vivo* experiments are needed to validate these targets to advance these potential targets as therapeutic options in the clinic.

## Methods

### Study population

This study of pediatric central nervous system tumors was approved by the Institutional Review Board Study #00030211. Tumor and non-tumor tissues were collected from patients treated at Dartmouth Hitchcock Medical Center from 1993 to 2017. Patients consented to use of tissues for research purposes. Histopathologic tumor type and grade for each sample were re-reviewed according to the 2021 WHO classification of CNS tumors and categorized into the major tumor types^5^. Tumor types included in this study are astrocytoma, embryonal tumors, ependymoma, glioneuronal/neuronal tumors, glioblastoma, and Schwannoma. The average age at diagnosis of subjects from whom the tumor tissues were derived from in this study was 9.3 (range: 0.75 – 18). Male subjects accounted for 62.9% of the tumor samples and female subjects accounted for 37.1% of the tumor samples. Non-tumor brain tissues were obtained from pediatric patients with epilepsy who underwent surgical resection. The average age at diagnosis of subjects from whom the non-tumor samples were derived from was 6.2 (0.58 – 11). Male subjects accounted for 33.3% of the non-tumor samples and female subjects accounted for 66.7% of the non-tumor samples. Specific demographic characteristics of patients for the study are provided in **Table 1** and sample information for each subject are provided in **Supplementary Table 1.**

### Identification of genetic variation with bulk tissue RNA-seq

RNA was collected using Qiagen RNeasy plus kit (Catalog ID: 74034, Qiagen, Hilden, Germany). RNA-seq libraries were prepared following the Takara Pico v3 low input protocol and sequenced on Illumina NextSeq500.

Raw RNA-seq data were trimmed for polyA sequences and low-quality bases using *cutadapt* (v2.4)^51^. Reads were aligned to human genome hg38 using *STAR*(v 2.7.2b)^52^. Duplicate read identification and other quality control checks for read alignment were performed using CollectRNASeqMetrics and MarkDuplicates in *Picard Tools*.^53^ Reads containing N were split using SplitNCigarReads function in the Genome Analysis Toolkit (GATK)^54,55^. Bases quality scores were recalibrated using known variants from the GATK resource bundle and with the BaseRecalibrator and ApplyBQSR functions in GATK^54,55^. Somatic SNV and indels were called with Mutect2 in tumor-only mode^54,55^. Only variants with at least read depth of 10, 5% allele frequency, read depth of 5 for the alternate allele were kept for analysis. The variants were then filtered for variants in sex or mitochondrial chromosomes, RNA editing sites, repeat masker regions, and variants in Panel of Normal (from GATK) references. Variants were then annotated using the Funcotator function in GATK^54,55^.

### Identification of copy number variation with DNA methylation arrays

DNA were treated with sodium bisulfite following the TrueMethyl^®^ oxBS Module (Tecan Genomics Inc, Redwood City, CA). Converted DNA were hybridized to Infinium HumanMethylationEPIC BeadChips. Raw idat files from the EPIC arrays were processed using preprocessNoob function in *minfi* in R^56^. Copy number variations of tumor samples were estimated in comparison to non-tumor samples using the CNV.fit function in *conumee* package in R^57^.

### Nuclei isolation, sample multiplexing, and single nuclei RNA-sequencing

Nuclei from fresh frozen tissues were isolated following the Nuclei Pure Prep nuclei isolation kit (Sigma-Aldrich, Catalog ID: NUC201) with some modifications. To summarize, ~10mg of tissue were washed with PBS to remove extraneous OCT the samples were frozen in. The tissue was homogenized with both wide and narrow pestles submerged in 2.5mL of the lysis buffer in a Dounce homogenizer. The lysate mixed with 4.5mL 1,8M sucrose cushion were gently layered on top of the 2.5mL of 1.8M sucrose cushion in Beckman ultracentrifuge tubes. Samples were centrifuged for 45 min at 13,000 RPM at 4°C in an ultracentrifuge. Samples were multiplexed with lipid-tagged oligonucleotides following the MULTI-seq protocol^20^. Nuclei were resuspended in 1% BSA PBS and filtered with 70um and 40um Flowmi filters. Nuclei were quantified with Cellometer K2 (Nexcelom, Lawrence, MA). We aimed for 2,500 – 5,000 nuclei per sample to be sequenced.

Libraries for single nuclei RNA-seq were prepared following the 10x Genomics Single Cell Gene Expression workflows (10x Genomics, Pleasanton, CA) and were sequenced on Illumina NextSeq500 to average 45,000 reads per cell. 10X Cell Ranger software was used to align sequences to GRch38 pre-mRNA reference genome and generate feature-barcode matrices for downstream analyses.

### Pre-processing snRNA-seq data

To filter low quality nuclei, only those with greater than 200 and less than 10,000 features and less than 5% of reads that map to the mitochondrial genes were used in downstream analyses. Pooled nuclei were demultiplexed by hashtag oligonucleotides using HTODemux function in Seurat v4^58-61^. Pooled samples were also demultiplexed using Vireo, a genotype based demultiplexing method^62^. We performed genetic demultiplexing analysis using genotype data following the methods described in Weber et al.^63^, implemented in a Nextflow workflow^64^. Briefly, bulk RNA-seq reads from each sample were mapped to the reference genome (GRCh38.p13) using STAR^52^. Pooled single-nuclei RNA-seq reads were mapped to the reference genome using STARsolo^65^. Variants among the samples within each pool were identified and genotyped with bcftools mpileup^66^ using the mapped bulk reads. Individual cells were then genotyped only at the sites identified using the bulk RNA using cellsnp-lite (mode 1a)^67^. Cell genotypes were used to identify the sample of origin for each cell using Vireo^62^. Code for the genetic demultiplexing workflow can be found at https://github.com/AlexsLemonade/alsf-scpca/tree/main/workflows/genetic-demux.

To integrate the methods, we first used sample identity assigned from the hashtag oligonucleotides. If the nuclei were confidently assigned a sample, it was compared to the genotype-based sample assignment. Those that did not match the same sample were filtered out. If the nuclei were assigned as a doublet or to none of the samples, the nuclei were assigned to a sample based on the genotype-based approach. 84,700 nuclei with confident sample assignment were used in analysis.

As our dataset included a very large number of nuclei to be integrated and was expected to have certain cell types only present in certain samples, we used the reciprocal PCA integration approach on the 2,000 most variable features to combine the nuclei from each sample. We first found the integration anchors with the FindIntegrationAnchors function then used the IntegrateData function in Seurat v4 to integrate all our filtered nuclei^59-61^.

### Dimension reduction and clustering of snRNA-seq data

The integrated dataset was scaled using the ScaleData function in Seurat. First, PCA dimensionality reduction analyses were done to identify 100 principal components (PCs). To further reduce the dimensionality and cluster our nuclei by their gene expression profile, we conducted UMAP analyses on the 50 PCs with highest standard deviation with RunUMAP function in Seurat^58,68^. Then, we clustered our cells using FindNeighbors (n_neighbors = 30) and FindClusters (resolution = 1.0) function in Seurat^58^.

### Gene set enrichment testing

Gene set enrichment tests at the single cell level were conducted using the Variance-Adjusted Mahalanobis (VAM) method^21^. The vamForSeurat function from the VAM R package was used to calculate enrichment scores for each nucleus. Brain cell type specific gene sets from the Molecular Signatures Database (MSigDB) v7.5.1 were used to validate our single cell identities^23-28^. For identifying cell types, p-values were calculated from the cumulative distribution function values generated by VAM. Nuclei were considered to be associated with a specific brain cell type-pathway if the VAM-generated p-value was ≤ 0.05. Nucleus-level pathway scoring was also conducted using VAM for pathways in the MsigDB Pathways Interaction Database (PID) collection^31^. PID Pathways were considered to be enriched in each nucleus at the FDR adjusted p-value threshold of 0.1 for the VAM-generated p-values.

Stemness scores for each nucleus were calculated using the stemness-associated gene list from Tirosh et al^29^ and the AddModuleScores function in Seurat.

### Differential gene expression and pathways

Differential expression analysis between tumor nuclei and non-tumor nuclei were conducted using monocle3^69-72^. Differential expression analyses were conducted only on the top 4,000 most variable features identified from the FindVariableFeatures Seurat function. The unadjusted differential expression testing was done using the fit_models function in monocle3 (v1.0.0) R package with the quasi-poisson distribution with the non-tumor nuclei being the referent gene expression profile^69,71,72^. The adjusted differential expression testing was done with the same quasi-poisson distribution with non-tumor nuclei being the referent but including the major cell type identity in the model. Gene types for each gene used in the differential expression testing were annotated using the org.Hs.eg.db^73^, Human genome annotation package, and mapIds function in the AnnotationDbi R package^74^. Pathways associated with the differentially expressed genes were identified using the Reactome pathways and ReactomePA R package^37^.

Pathways important for each cell cluster were identified using FindAllMarkers function in Seurat v4 with the Wilcoxon rank sum test in Seurat on the binary classification of PID pathways enrichment for each nuclei^75^. Log fold change and minimum percentage of cells enriched in each pathway were both set to 0. To identify the pathways with greater number of nuclei with enriched pathway per cluster, we selected pathways that were only positive in direction in the FindAllMarkers options.

### Statistical testing

Observed proportion of genes that were either increased or decreased in the same direction in the shared differentially expressed genes among all the tumor types (60.9%) were compared to expected proportion of genes that would be increased or decreased in the same direction across all the tumor types (3.13%) using a one-sample proportion test. The expected proportions were determined based on the permutations of direction of change compared to non-tumor for the six tumor types.

## References

[1] Siegel R. L., Miller K. D., Fuchs H. E. & Jemal A. Cancer statistics, 2022. Ca Cancer J Clin 72, 7–33 (2022). 35020204 10.3322/caac.21708

[2] Ostrom Q. T., Cioffi G., Waite K., Kruchko C. & Barnholtz-Sloan J. S. CBTRUS Statistical Report: Primary Brain and Other Central Nervous System Tumors Diagnosed in the United States in 2014–2018. Neuro-oncology 23, iii1–iii105 (2021). 34608945 10.1093/neuonc/noab200 PMC8491279

[3] Friedman D. L. Subsequent Neoplasms in 5-Year Survivors of Childhood Cancer: The Childhood Cancer Survivor Study. Jnci J National Cancer Inst 102, 1083–1095 (2010). 10.1093/jnci/djq238 PMC2907408 20634481

[4] Tsui K. Subsequent neoplasms in survivors of childhood central nervous system tumors: risk after modern multimodal therapy. Neuro-oncology 17, 448–456 (2015). 25395462 10.1093/neuonc/nou279 PMC4483102

[5] Louis D. N. The 2021 WHO Classification of Tumors of the Central Nervous System: a summary. Neuro-oncology 23, 1231–1251 (2021). 34185076 10.1093/neuonc/noab106 PMC8328013

[6] Northcott P. A. The whole-genome landscape of medulloblastoma subtypes. Nature 547, 311–317 (2017). 28726821 10.1038/nature22973 PMC5905700

[7] Hovestadt V. Resolving medulloblastoma cellular architecture by single-cell genomics. Nature 572, 74–79 (2019). 31341285 10.1038/s41586-019-1434-6 PMC6754173

[8] Taylor M. D. Molecular subgroups of medulloblastoma: the current consensus. Acta Neuropathol 123, 465–472 (2012). 22134537 10.1007/s00401-011-0922-z PMC3306779

[9] Northcott P. A. Subgroup-specific structural variation across 1,000 medulloblastoma genomes. Nature 488, 49–56 (2012). 22832581 10.1038/nature11327 PMC3683624

[10] Lin C. Y. Active medulloblastoma enhancers reveal subgroup-specific cellular origins. Nature 530, 57–62 (2016). 26814967 10.1038/nature16546 PMC5168934

[11] Pajtler K. W. Molecular Classification of Ependymal Tumors across All CNS Compartments, Histopathological Grades, and Age Groups. Cancer Cell 27, 728–743 (2015). 25965575 10.1016/j.ccell.2015.04.002 PMC4712639

[12] Parker M. C11orf95–RELA fusions drive oncogenic NF-κB signalling in ependymoma. Nature 506, 451–455 (2014). 24553141 10.1038/nature13109 PMC4050669

[13] Doz F. Efficacy and safety of larotrectinib in TRK fusion-positive primary central nervous system tumors. Neuro-oncology 24, noab274- (2021). 10.1093/neuonc/noab274 PMC9159442 34850167

[14] FDA approves larotrectinib for solid tumors with NTRK gene fusions ∣ FDA. https://www.fda.gov/drugs/fda-approves-larotrectinib-solid-tumors-ntrk-gene-fusions.

[15] Filbin M. G. Developmental and oncogenic programs in H3K27M gliomas dissected by single-cell RNA-seq. Science 360, 331–335 (2018). 29674595 10.1126/science.aao4750 PMC5949869

[16] Gojo J. Single-Cell RNA-Seq Reveals Cellular Hierarchies and Impaired Developmental Trajectories in Pediatric Ependymoma. Cancer Cell 38, 44–59.e9 (2020). 32663469 10.1016/j.ccell.2020.06.004 PMC7479515

[17] Gillen A. E. Single-Cell RNA Sequencing of Childhood Ependymoma Reveals Neoplastic Cell Subpopulations That Impact Molecular Classification and Etiology. Cell Reports 32, 108023 (2020). 32783945 10.1016/j.celrep.2020.108023 PMC7452755

[18] Carter M. Genetic abnormalities detected in ependymomas by comparative genomic hybridisation. Brit J Cancer 86, 929–939 (2002). 11953826 10.1038/sj.bjc.6600180 PMC2364143

[19] Rajeshwari M. Evaluation of chromosome 1q gain in intracranial ependymomas. J Neuro-oncol 127, 271–278 (2016). 10.1007/s11060-015-2047-z 26725097

[20] McGinnis C. S. MULTI-seq: sample multiplexing for single-cell RNA sequencing using lipid-tagged indices. Nat Methods 16, 619–626 (2019). 31209384 10.1038/s41592-019-0433-8 PMC6837808

[21] Frost H. R. Variance-adjusted Mahalanobis (VAM): a fast and accurate method for cell-specific gene set scoring. Nucleic Acids Res 48, gkaa582- (2020). 10.1093/nar/gkaa582 PMC7498348 32633778

[22] Subramanian A. Gene set enrichment analysis: A knowledge-based approach for interpreting genome-wide expression profiles. Proc National Acad Sci 102, 15545–15550 (2005). 10.1073/pnas.0506580102 PMC1239896 16199517

[23] Liberzon A. Molecular signatures database (MSigDB) 3.0. Bioinformatics 27, 1739–1740 (2011). 21546393 10.1093/bioinformatics/btr260 PMC3106198

[24] Liberzon A. The Molecular Signatures Database Hallmark Gene Set Collection. Cell Syst 1, 417–425 (2015). 26771021 10.1016/j.cels.2015.12.004 PMC4707969

[25] Fan X. Spatial transcriptomic survey of human embryonic cerebral cortex by single-cell RNA-seq analysis. Cell Res 28, 730–745 (2018). 29867213 10.1038/s41422-018-0053-3 PMC6028726

[26] Zhong S. A single-cell RNA-seq survey of the developmental landscape of the human prefrontal cortex. Nature 555, 524–528 (2018). 29539641 10.1038/nature25980

[27] Cao J. A human cell atlas of fetal gene expression. Science 370, (2020). 10.1126/science.aba7721 PMC7780123 33184181

[28] Manno G. L. Molecular Diversity of Midbrain Development in Mouse, Human, and Stem Cells. Cell 167, 566–580.e19 (2016). 27716510 10.1016/j.cell.2016.09.027 PMC5055122

[29] Tirosh I. Single-cell RNA-seq supports a developmental hierarchy in human oligodendroglioma. Nature 539, 309–313 (2016). 27806376 10.1038/nature20123 PMC5465819

[30] Zhao W., Li Y. & Zhang X. Stemness-related markers in cancer. Cancer Transl Medicine 3, 87 (2017). 10.4103/ctm.ctm_69_16 PMC5737740 29276782

[31] Schaefer C. F. PID: the Pathway Interaction Database. Nucleic Acids Res 37, D674–D679 (2009). 18832364 10.1093/nar/gkn653 PMC2686461

[32] Kuhn A. Cell population-specific expression analysis of human cerebellum. Bmc Genomics 13, 610 (2012). 23145530 10.1186/1471-2164-13-610 PMC3561119

[33] Fridman W. H., Pagès F., Sautès-Fridman C. & Galon J. The immune contexture in human tumours: impact on clinical outcome. Nat Rev Cancer 12, 298–306 (2012). 22419253 10.1038/nrc3245

[34] Consortium*, T. S. The Tabula Sapiens: A multiple-organ, single-cell transcriptomic atlas of humans. Science 376, eabl4896 (2022). 35549404 10.1126/science.abl4896 PMC9812260

[35] Egeblad M., Nakasone E. S. & Werb Z. Tumors as Organs: Complex Tissues that Interface with the Entire Organism. Dev Cell 18, 884–901 (2010). 20627072 10.1016/j.devcel.2010.05.012 PMC2905377

[36] Kaur N. Wnt3a mediated activation of Wnt/β-catenin signaling promotes tumor progression in glioblastoma. Mol Cell Neurosci 54, 44–57 (2013). 23337036 10.1016/j.mcn.2013.01.001

[37] Yu G. & He Q.-Y. ReactomePA: an R/Bioconductor package for reactome pathway analysis and visualization. Mol Biosyst 12, 477–479 (2015). 10.1039/c5mb00663e 26661513

[38] Johnson R. A. Cross-species genomics matches driver mutations and cell compartments to model ependymoma. Nature 466, 632–636 (2010). 20639864 10.1038/nature09173 PMC2912966

[39] Taylor M. D. Radial glia cells are candidate stem cells of ependymoma. Cancer Cell 8, 323–335 (2005). 16226707 10.1016/j.ccr.2005.09.001

[40] Reitman Z. J. Mitogenic and progenitor gene programmes in single pilocytic astrocytoma cells. Nat Commun 10, 3731 (2019). 31427603 10.1038/s41467-019-11493-2 PMC6700116

[41] Abou-Antoun T. J., Hale J. S., Lathia J. D. & Dombrowski S. M. Brain Cancer Stem Cells in Adults and Children: Cell Biology and Therapeutic Implications. Neurotherapeutics 14, 372–384 (2017). 28374184 10.1007/s13311-017-0524-0 PMC5398995

[42] Castelo-Branco P. & Tabori U. Promises and challenges of exhausting pediatric neural cancer stem cells. Pediatr Res 71, 523–528 (2012). 22430389 10.1038/pr.2011.63

[43] Kong D.-S. Cancer Stem Cells in Brain Tumors and Their Lineage Hierarchy. Int J Stem Cells 5, 12–15 (2012). 24298350 10.15283/ijsc.2012.5.1.12 PMC3840978

[44] Bao S. Glioma stem cells promote radioresistance by preferential activation of the DNA damage response. Nature 444, 756–760 (2006). 17051156 10.1038/nature05236

[45] Hemmati H. D. Cancerous stem cells can arise from pediatric brain tumors. Proc National Acad Sci 100, 15178–15183 (2003). 10.1073/pnas.2036535100 PMC299944 14645703

[46] Singh S. K. Identification of human brain tumour initiating cells. Nature 432, 396–401 (2004). 15549107 10.1038/nature03128

[47] Galli R. Isolation and Characterization of Tumorigenic, Stem-like Neural Precursors from Human Glioblastoma. Cancer Res 64, 7011–7021 (2004). 15466194 10.1158/0008-5472.CAN-04-1364

[48] Lou C. H. Posttranscriptional Control of the Stem Cell and Neurogenic Programs by the Nonsense-Mediated RNA Decay Pathway. Cell Reports 6, 748–764 (2014). 24529710 10.1016/j.celrep.2014.01.028 PMC3962089

[49] Jaffrey S. R. & Wilkinson M. F. Nonsense-mediated RNA decay in the brain: emerging modulator of neural development and disease. Nat Rev Neurosci 19, 715–728 (2018). 30410025 10.1038/s41583-018-0079-z PMC6396682

[50] Jolly L. A., Homan C. C., Jacob R., Barry S. & Gecz J. The UPF3B gene, implicated in intellectual disability, autism, ADHD and childhood onset schizophrenia regulates neural progenitor cell behaviour and neuronal outgrowth. Hum Mol Genet 22, 4673–4687 (2013). 23821644 10.1093/hmg/ddt315

[51] Martin M. Cutadapt removes adapter sequences from high-throughput sequencing reads. Embnet J 17, 10–12 (2011).

[52] Dobin A. STAR: ultrafast universal RNA-seq aligner. Bioinformatics 29, 15–21 (2013). 23104886 10.1093/bioinformatics/bts635 PMC3530905

[53] Institute B. Picard Toolkit. https://broadinstitute.github.io/picard/ (2019).

[54] Auwera G. V. der & O’Connor B. Genomics in the Cloud: Using Docker, GATK, and WDL in Terra. (O’Reilly Media, 2020).

[55] McKenna A. The Genome Analysis Toolkit: a MapReduce framework for analyzing next-generation DNA sequencing data. Genome Research 20, 1297–1303 (2010). 20644199 10.1101/gr.107524.110 PMC2928508

[56] Aryee M. J. Minfi: a flexible and comprehensive Bioconductor package for the analysis of Infinium DNA methylation microarrays. Bioinformatics 30, 1363–1369 (2014). 24478339 10.1093/bioinformatics/btu049 PMC4016708

[57] Hovestadt V. & Zapatka M. conumee: Enhanced copy-number variation analysis using Illumina DNA methylation arrays. (R package version 1.9.0). 10.1093/bioinformatics/btae029 PMC10868300 38244574

[58] Satija R., Farrell J. A., Gennert D., Schier A. F. & Regev A. Spatial reconstruction of single-cell gene expression data. Nat Biotechnol 33, 495–502 (2015). 25867923 10.1038/nbt.3192 PMC4430369

[59] Hao Y. Integrated analysis of multimodal single-cell data. Cell 184, 3573–3587.e29 (2021). 34062119 10.1016/j.cell.2021.04.048 PMC8238499

[60] Stuart T. Comprehensive Integration of Single-Cell Data. Cell 177, 1888–1902.e21 (2019). 31178118 10.1016/j.cell.2019.05.031 PMC6687398

[61] Butler A., Hoffman P., Smibert P., Papalexi E. & Satija R. Integrating single-cell transcriptomic data across different conditions, technologies, and species. Nat Biotechnol 36, 411–420 (2018). 29608179 10.1038/nbt.4096 PMC6700744

[62] Huang Y., McCarthy D. J. & Stegle O. Vireo: Bayesian demultiplexing of pooled single-cell RNA-seq data without genotype reference. Genome Biol 20, 273 (2019). 31836005 10.1186/s13059-019-1865-2 PMC6909514

[63] Weber L. M. Genetic demultiplexing of pooled single-cell RNA-sequencing samples in cancer facilitates effective experimental design. Gigascience 10, giab062- (2021). 34553212 10.1093/gigascience/giab062 PMC8458035

[64] Tommaso P. D. Nextflow enables reproducible computational workflows. Nat Biotechnol 35, 316–319 (2017). 28398311 10.1038/nbt.3820

[65] Kaminow B., Yunusov D. & Dobin A. STARsolo: accurate, fast and versatile mapping/quantification of single-cell and single-nucleus RNA-seq data. Biorxiv 2021.05.05.442755 (2021) doi:10.1101/2021.05.05.442755.

[66] Danecek P. Twelve years of SAMtools and BCFtools. Gigascience 10, giab008 (2021). 33590861 10.1093/gigascience/giab008 PMC7931819

[67] Huang X. & Huang Y. Cellsnp-lite: an efficient tool for genotyping single cells. Bioinformatics 37, 4569–4571 (2021). 10.1093/bioinformatics/btab358 33963851

[68] McInnes L., Healy J. & Melville J. UMAP: Uniform Manifold Approximation and Projection for Dimension Reduction. Arxiv (2018) doi:10.48550/arxiv.1802.03426.

[69] Trapnell C. The dynamics and regulators of cell fate decisions are revealed by pseudotemporal ordering of single cells. Nat Biotechnol 32, 381–386 (2014). 24658644 10.1038/nbt.2859 PMC4122333

[70] Qiu X. Reversed graph embedding resolves complex single-cell trajectories. Nat Methods 14, 979–982 (2017). 28825705 10.1038/nmeth.4402 PMC5764547

[71] Cao J. The single-cell transcriptional landscape of mammalian organogenesis. Nature 566, 496–502 (2019). 30787437 10.1038/s41586-019-0969-x PMC6434952

[72] Qiu X. Single-cell mRNA quantification and differential analysis with Census. Nat Methods 14, 309–315 (2017). 28114287 10.1038/nmeth.4150 PMC5330805

[73] Carlson M. org.Hs.eg.db: Genome wide annotation for Human. (R package version 3.8.2.).

[74] Pagès H., Carlson M., Falcon S. & Li N. AnnotationDbi: Manipulation of SQLite-based annotations in Bioconductor. (R package version 1.60.0).

[75] Finak G. MAST: a flexible statistical framework for assessing transcriptional changes and characterizing heterogeneity in single-cell RNA sequencing data. Genome Biol 16, 278 (2015). 26653891 10.1186/s13059-015-0844-5 PMC4676162

